# A study of the association of rs12040273 with susceptibility and severity of coronary artery disease in a Chinese Han population

**DOI:** 10.1186/s12872-018-0743-2

**Published:** 2018-01-19

**Authors:** Bo Yang, Shan Yan, Jianjun Yan, Yafei Li, Mohammad Reeaze Khurwolah, Liansheng Wang, Zhong Chen

**Affiliations:** 1 Department of Cardiology, the First Affiliated Hospital of Nanjing Medical University, Nanjing, 210029 China; 2 Department of Cardiology, Affiliated Sixth People’s Hospital East, Shanghai University of Medicine and Health Sciences; Shanghai Jiao Tong University Affiliated Sixth People’s Hospital East, No. 222 Huanhu Xisan Road, Shanghai, 201306 China

**Keywords:** Single nucleotide polymorphisms (SNPs), rs12040273, Susceptibility, Coronary artery disease

## Abstract

**Background:**

The single nucleotide polymorphism (SNP) rs12040273, a variant of UDP-N–acetylgalactosamine, polypeptide GalNAc-transferase 2, has recently been reported to be significantly associated with development of carotid artery intima-media thickness (IMT) in a Chinese population based on a genome-wide association study. Because IMT is a potent marker of coronary artery disease (CAD), the aim of this study was to evaluate the relation of rs12040273 to susceptibility and severity of CAD in a Chinese Han population.

**Methods:**

We performed a hospital-based case-control study. Three hundred and thirty-one individuals (199 CAD patients and 112 non-CAD controls) undergoing coronary angiography were consecutively enrolled in the study. The Gensini score results were used to assess the severity of CAD. The method of polymerase chain reaction-ligase detection reaction (PCR-LDR) was used to distinguish different genotypes at rs12040273.

**Results:**

The distribution of genotypes at rs12040273 was comparable between CAD patients and non-CAD controls (*P* > 0.05). The frequencies of the genotypes were also not significantly associated with the risk of CAD and its severity assessed by the Gensini score method, with the OR of 1.38 (95% CI = 0.80–2.40, *P* = 0.24) and 1.14 (95% CI = 0.69–1.86, *P* = 0.60) respectively. However, stratified analysis showed that the serum HDL-C levels of subjects with the CC genotype were significantly higher than those with CT/TT genotypes in non-CAD controls (*P* = 0.002).

**Conclusion:**

Our results suggest that the rs12040273 variants might not be associated with the susceptibility of CAD or its severity in a Chinese Han population. Moreover, the CC genotype could be associated with elevated serum HDL-C levels.

## Background

Coronary artery disease (CAD) remains a major cause of morbidity and mortality worldwide, although its effective therapeutic strategies have been greatly improved in recent years. Atherosclerosis plays a vital role in the pathological process of CAD, which comprises multiple risk factors and clinical entities that include genetic and nongenetic aspects, and is also affected by their interactions [1].

As a member of a homologous family of UDP-N–acetylgalactosamine, polypeptide GalNAc-transferase2 (GALNT2) is located at chromosome 1q41-q42, which shares overall amino acid sequence similarities of approximately 45–50% and has been found to span 16 exons [2]. GALNT2 encodes an enzyme, N-acetylglucosaminyltransferase 2, which is involved in the process of O-linked glycosylation of proteins. GALNT2 has been identified as a candidate gene in lipid metabolism by genome-wide association studies (GWAS), and its single nucleotide polymorphisms (SNPs) might be correlated with plasma lipids [3, 4]. Lipid disorders are closely related to the formation of coronary artery lesions and are independent factors in the prediction and prognosis of CAD [5].

More recently, the Xie et al. study revealed that rs12040273 is remarkably associated with carotid artery intima-media thickness (IMT) at the genome level, based on GWAS on progression of IMT in a Chinese cohort over 10 years [6]. With atherosclerosis as the common pathophysiologic mechanism, both CAD and carotid IMT share similar risk factors, including hypertension, diabetes, dyslipidemia, and metabolic syndrome [7]. Moreover, carotid artery IMT has been a potent marker of asymptomatic subclinical atherosclerosis [8]. In combination, these findings suggest that rs12040273 might potentially be associated with the risk of CAD.

However, no study has previously investigated whether rs12040273 might be linked to the susceptibility and severity of CAD. Therefore, we performed this hospital-based case-control study to explore whether rs12040273 might be associated with risk and severity of CAD in a Chinese Han population, by which optimal preventive and therapeutic strategies to reduce the heavy burden of morbidity and mortality from CAD could be further improved.

## Methods

### Study population

A total of 311 consecutive patients of Chinese Han ethnicity undergoing coronary angiography (CAG) from January 2014 to December 2015 admitted to the Department of Cardiology, Shanghai Jiao Tong University Affiliated Sixth People’s Hospital East were included in the study. One hundred and ninety-nine patients with confirmed CAD acted as cases and 112 non-CAD subjects as controls. CAD was defined as coronary artery stenosis ≥50% in at least one main vessel or its major branches, and the participants with all main coronary arteries and large branches narrowing less than 30% were enrolled as non-CAD controls. The Gensini score method was used to evaluate the severity of CAD [9]. Stenosis severity of <25%, 26%–50%, 51%–75%, 76%–90%, 91%–99%, and total occlusion were given a Gensini score of 1, 2, 4, 8, 16, and 32, respectively [9]. The CAD patients were divided into high (> 40 points) and low (≤ 40 points) subgroups, according to Gensini scores [10]. The exclusion criteria included severe liver or kidney disease, heparin use or bleeding disorders, thyroid disease, and cardiomyopathy and malignant diseases.

### Ethics approval and consent to participate

The Ethics Committee of Shanghai Jiao Tong University Affiliated Sixth People’s Hospital East approved this study, and all participants gave their written consent before the study began.

### Definitions of baseline risk factors and measurement

All conventional CAD risk factors including hypertension, cigarette smoking, dyslipidemia, and diabetes were defined as reported before [11]. Fasting blood sugar (FBS), total cholesterol (TC), triglycerides (TG), HDL-C, and low-density lipoprotein cholesterol (LDL-C) were measured and calculated using standard methods.

### DNA extraction and genotyping

Lymphocytic DNA samples were drawn from peripheral venous blood of all participants by using the available AxyPrep DNA Blood kit (Axygen Scientific Inc., CA, USA). The rs12040273 polymorphism was genotyped by the modified method of polymerase chain reaction-ligase detection reaction (PCR-LDR), as previously described [12]. Five percent of samples were selected randomly for sequencing to confirm the results of genotyping.

### Statistical analyses

Statistical analyses were conducted using SPSS 19.0 for Windows (SPSS Inc., Chicago, USA). Continuous variables and categorical variables are described as mean ± standard deviation and numbers or percentages as appropriate. A general linear model was performed to adjust for the effects of age and sex. Other data expressed as frequencies and percentages were analyzed using the chi-square (χ2)-test, which was also used to compare the allele frequencies and genotype distributions between non-CAD controls and CAD cases. The association between the genotypes and risk of CAD was assessed by calculating values for odds ratios (ORs) and 95% confidence intervals (95% CIs). Logistic regression was used to adjust for covariates including hypertension, diabetes, smoking status, age, sex, and dyslipidemia. The linear trend in the association of rs12040273 polymorphism with the severity of CAD was evaluated by the χ2-test for trends. Two-tailed *P*-value less than 0.05 was considered statistically significant.

## Results

### Demographic information

The characteristics of CAD subjects and controls are shown in Table 1. CAD patients were older, and a large percentage of them were male smokers. Moreover, a higher proportion of patients with CAD were affected by hypertension, diabetes, and dyslipidemia compared with the control group (all *P* < 0.05). The serum HDL-C level in the CAD group was lower in contrast to that in the control group (*P* = 0.002), whereas no significant differences were found with regards to serum TC, LDL-C, and TG levels between the 2 groups (all *P* > 0.05).

**Table 1:** The characteristics of CAD patients and non-CAD controls

Characteristics	Non-CAD controls (*n* = 112)	CAD cases (*n* = 199)	*P*
Age (years)	59.69 ± 11.81	67.44 ± 11.97	< 0.001
Sex (male), n (%)	43 (38.4.4)	126 (63.3)	< 0.001
Hypertension, n (%)	67 (59.8)	143 (71.9)	0.03
Diabetes, n (%)	17 (15.2)	63 (31.7)	0.001
Dyslipidemia, n (%)	50(44.6)	116 (58.3)	0.021
Smoking, n (%)	20 (17.9)	70 (35.2)	0.001
TC (mmol/L)	4.46 ± 1.01	4.39 ± 1.35	0.63
TG (mmol/L)	1.50 ± 0.99	1.65 ± 2.02	0.18
HDL-C (mmol/L)	1.26 ± 0.31	1.16 ± 0.26	0.002
LDL-C (mmol/L)	2.58 ± 0.66	2.51 ± 0.79	0.42

*HDL-C* high-density lipoprotein cholesterol, *LDL-C* low-density lipoprotein cholesterol, *TC* total cholesterol, *TG* triglyceride

Age, TC, LDL-C, HDL-C, TG and glucose (expressed as mean ± SD) were abnormally distributed and analyzed by Mann-Whitney U-test. Other data are expressed as frequencies and percentages and evaluated by χ2-test

### Genotypes, allele frequencies of rs12040273, and their associations with risk of CAD

The minor allele T frequency of rs12040273 equals 0.362, and the genotype distribution in the present study meets with the Hardy-Weinberg equilibrium (*P* > 0.05). The frequencies of the CC, CT, and CT genotypes were 45.5%, 37.5%, and 17.0% in the controls, and 38.2%, 46.2%, and 15.6% in the CAD patients, respectively (Table 2). Distribution of rs12040273 genotypes was comparable between the CAD group and controls (*P* > 0.05). The risk estimates for the variants at rs12040273 among CAD patients and controls were listed in Table 2. Furthermore, after adjusting for CAD risk factors including hypertension, diabetes, dyslipidemia, sex, and age, the OR for subjects with the genotypes CT and TT was 1.38 (95% CI: 0.80–2.40, *P* = 0.24).

**Table 2:** Distribution and the risk estimates of rs12040273 with CAD

		Non-CAD controls	CAD cases	Crude	P	Adjusted^a^	P
		(*n* = 112)	(*n* = 199)	OR (95% CI)		OR (95% CI)	
	Genotypes, n (%)						
Rs12040273	CC	51 (45.5)	76 (38.2)	1.00		1.00	
	CT	42 (37.5)	92 (46.2)	1.47 (0.88–2.44)	0.13	1.54 (0.86–2.78)	0.14
	TT	19(17.0)	31(15.6)	1.09 (0.56–2.14)	0.79	1.01 (0.45–2.26)	0.98
	CT + TT	61 (54.4)	123 (61.8)	1.35 (0.84–2.16)	0.14	1.38 (0.80–2.40)	0.24

^a^Adjusted for hypertension, diabetes, dyslipidemia, smoking status, sex and age

*CI* confidence interval, *OR* odds ratio

### The association of genotypes of rs 12,040,273 with CAD severity

Table 3 shows the association between the polymorphisms studied and the severity of CAD expressed by the Gensini score. The distribution of different genotypes was similar between the subgroups of low and high Gensini scores (*P* > 0.05). Additionally, no significant association was found between the examined polymorphisms and the Gensini scores after adjusting for the covariates of age, sex, hypertension, diabetes, and dyslipidemia (adjusted OR = 1.14, 95% CI: 0.69–1.86, *P* = 0.60).

**Table 3:** Association of rs12040273 variants with Gensini scores

SNPs	Groups	Genotype	Crude OR(95%CI)	P	Adjusted^a^ OR(95%CI)	P
Rs12040273		CC/CT/TT				
	Control	51/42/19	1		1	
	Gensini< 40	52/56/22	1.11 (0.78–1.60)	0.55	1.09 (0.73–1.64)	0.66
	Gensini> 40	24/36/9	1.14 (0.75–1.74)	0.41	1.14 (0.69–1.86)	0.60

^a^Adjusted for hypertension, diabetes, dyslipidemia, smoking status, sex and age

*CI* confidence interval, *OR* odds ratio

### Lipid profiles in CAD cases and controls with different rs12040273 genotypes

The HDL-C levels were significantly different between the CC and CT/TT genotypes in the control group (*P* = 0.002), but not in the CAD patients (Table 4). The controls with a CC genotype had an increased serum HDL-C level compared with those with a CT/TT genotype. However, we could not find a significant association between the rs12040273 genotypes and TG, TC, and LDL-C levels between the two groups.

**Table 4:** Lipid profiles with rs12040273 genotypes between CAD cases and controls

Characteristics	Non-CAD controls		P	CAD Cases		P
	CC	CT + TT		CC	CT + TT	
TG (mmol/L)	1.35 ± 0.59	1.62 ± 1.22	0.15	1.53 ± 0.89	1.73 ± 2.98	0.15
TC(mmol/L)	4.51 ± 1.08	4.43 ± 0.97	0.66	4.28 ± 1.33	4.46 ± 1.36	0.36
HDL-C (mmol/L)	1.35 ± 0.35	1.18 ± 0.24	0.002	1.14 ± 0.27	1.17 ± 0.24	0.39
LDL-C (mmol/L)	2.64 ± 0.70	2.53 ± 0.63	0.36	2.45 ± 0.78	2.55 ± 0.80	0.43

*TG* triglyceride; *TC* total cholesterol; *HDL-C* high-density lipoprotein cholesterol, *LDL-C* low-density lipoprotein cholesterol

TG, TC, HDL-C and LDL-C (expressed as mean ± SD) were analyzed by General linear model adjust for age and sex

## Discussion

In this study, we found that the distribution of genotypes at rs12040273 was not remarkably different between the CAD patients and the non-CAD controls, and the disparity of its distribution between the high and low Gensini score groups also did not reach statistical significance. Importantly, our stratified analysis suggests that the rs12040273 C allele is associated with an increased serum HDL-C level in non-CAD controls, and it may be associated with HDL metabolism. Therefore, the results of our study are not consistent with the results of previous studies [3, 4, 13]. This lack of consistency suggests that the rs12040273 polymorphism might not be associated with increased susceptibility and severity of CAD in this Chinese Han population.

Studies have proved that variations in the first intron of the GALNT2 gene at 1q42 are associated with changes in TG and HDL-C levels [3, 4, 13–15]. In a population-based GWAS, 302,218 SNPs were genotyped in Chinese adults 53–79 years of age. Xie et al. found a significant association between rs12040273 and carotid artery IMT [6]. Increased IMT is an early marker of atherosclerosis and is an independent predictor of cardiac events [8, 16], and the formation on atherosclerotic plaques is the key pathological process for carotid artery disease and CAD, which also share common risk factors [17]. It is thus hypothesized that rs12040273 could potentially be associated with the risk and severity of CAD, although no exact genetic evidence of this correlation has been reported. The Gensini score is an appropriate evaluation method for the severity of CAD [18] and therefore was used in our study.

Ping et al. examined the role of promoter methylation of the GALNT2 gene in a Chinese Han population and found that aging and smoking were important factors that might influence DNA promoter hypermethylation of GALNT2 [19]. Moreover, Marucci et al. studied the relation between hyperglycemia and GALNT2 down-regulation observed in human PWBC, and observed GALNT2 expression is reduced in patients with type 2 diabetes [20]. Therefore, we cannot exclude the underlying factors that influence the expression of rs12040273, because no significant differences existed in baseline clinical characteristics between the CAD and control groups. Taken together, to ascertain whether rs12040273 is a functional site, further studies related to its complex mechanism of genetics and molecular biology are needed, including metabolism disturbance, immune or inflammatory response, and rupture of atherosclerotic plaques.

Interestingly, our stratified analysis showed that the serum HDL-C levels in subjects with CC genotype are significantly higher than that of participants with a CT/TT genotype in the controls, although no significant interaction was detected between the genotypes and serum HDL-C level in the CAD group, and serum TC, LDL-C, and TG levels between the two groups. Holleboom et al. revealed a missense mutation in GALNT2 caused by a reduction in GalNAc-T2 catalytic activity and increased HDL-C levels in man [21]. Furthermore, Teslovich et al. found that reducing the transcript level of GALNT2 through the delivery of an shRNA via a vector could produce higher HDL-C levels compared with levels in the control group [13]. Studies have demonstrated that serum HDL-C not only plays a role in prevention of CAD but also is highly heritable, and heritability estimate is approximately 40–60% for HDL-C levels [22, 23]. To some extent, our study suggests that the rs12040273 C allele may be a protective factor for CAD due to its elevated serum HDL-C levels, whereas further studies should be done to explore its mechanism of genetic variation.

The present study has several limitations. First, selection bias may exist due to the study design, and the power of our study was reduced. Second, we could not obtain a comprehensive view of the rs12040273 genetic variability, because the indexes for assessing its function were relatively simple. Lastly, a single case–case study and relatively small sample sizes are too weak to fully explore the relationship between this SNP and its susceptibility to CAD and its severity.

## Conclusion

Our study shows that the rs12040273 polymorphism is not significantly associated with the risk and severity of CAD in a Chinese Han population. Interestingly, our stratified analysis suggests that the rs12040273 C allele is associated with an increased serum HDL-C level in non-CAD controls, and it may be involved in the metabolism of HDL-C. Studies with large samples are warranted to explore its genetic function and interaction with covariants, including inflammatory and environmental factors.

## Abbreviations

CAD: coronary artery disease
CAG: coronary angiography
CI: confidence interval
DM: diabetes mellitus
DNA: deoxyribonucleic acid
FBS: fasting blood sugar
GALNT2: GalNAc-transferase2
GWAS: genome-wide association studies
HDL-C: high-density lipoprotein cholesterol
IMT: intima-media thickness
LDL-C: low-density lipoprotein cholesterol
OR: odds ratio
PCR-LDR: polymerase chain reaction-ligase detection reaction
SNPs: single nucleotide polymorphisms
TC: total cholesterol
TG: triglycerides
